# p53 Binds Preferentially to Non-B DNA Structures Formed by the Pyrimidine-Rich Strands of GAA·TTC Trinucleotide Repeats Associated with Friedreich’s Ataxia

**DOI:** 10.3390/molecules24112078

**Published:** 2019-05-31

**Authors:** Robert Helma, Pavla Bažantová, Marek Petr, Matej Adámik, Daniel Renčiuk, Vlastimil Tichý, Alena Pastuchová, Zuzana Soldánová, Petr Pečinka, Richard P. Bowater, Miroslav Fojta, Marie Brázdová

**Affiliations:** 1 Institute of Biophysics, Academy of Sciences of the Czech Republic v.v.i., Královopolská 135, 612 65 Brno, Czech Republic; rhelma@ibp.cz (R.H.); pavla.bazantova@gmail.com (P.B.); zhlavek@ibp.cz (M.P.); matej@ibp.cz (M.A.); renciuk@ibp.cz (D.R.); vlastim.tichy@gmail.com (V.T.); 357864@mail.muni.cz (A.P.); babkovazuzana@gmail.com (Z.S.); Petr.Pecinka@osu.cz (P.P.); fojta@ibp.cz (M.F.); 2 Department of Molecular Biology and Pharmaceutical Biotechnology, Faculty of Pharmacy, University of Veterinary and Pharmaceutical Sciences Brno, Palackého 1/3, 612 42 Brno, Czech Republic; 3 Faculty of Science, University of Ostrava, Chittussiho 10, 701 03 Ostrava, Czech Republic; 4 School of Biological Sciences, University of East Anglia, Norwich Research Park, Norwich NR4 7TJ, UK; R.Bowater@uea.ac.uk; 5 Central European Institute of Technology, Masaryk University, Kamenice 753/5, CZ-62500 Brno, Czech Republic

**Keywords:** trinucleotide repeat, p53, non-B DNA, DNA hairpin, DNA–protein, frataxin

## Abstract

Expansions of trinucleotide repeats (TNRs) are associated with genetic disorders such as Friedreich’s ataxia. The tumor suppressor p53 is a central regulator of cell fate in response to different types of insults. Sequence and structure-selective modes of DNA recognition are among the main attributes of p53 protein. The focus of this work was analysis of the p53 structure-selective recognition of TNRs associated with human neurodegenerative diseases. Here, we studied binding of full length p53 and several deletion variants to TNRs folded into DNA hairpins or loops. We demonstrate that p53 binds to all studied non-B DNA structures, with a preference for non-B DNA structures formed by pyrimidine (Py) rich strands. Using deletion mutants, we determined the C-terminal DNA binding domain of p53 to be crucial for recognition of such non-B DNA structures. We also observed that p53 in vitro prefers binding to the Py-rich strand over the purine (Pu) rich strand in non-B DNA substrates formed by sequence derived from the first intron of the frataxin gene. The binding of p53 to this region was confirmed using chromatin immunoprecipitation in human Friedreich’s ataxia fibroblast and adenocarcinoma cells. Altogether these observations provide further evidence that p53 binds to TNRs’ non-B DNA structures.

## 1. Introduction

Wild type p53 (p53) is an important transcription factor that plays a significant role in the prevention of tumor cells development and is widely known as “the guardian of the genome and epigenome” [1,2]. As a response to potentially oncogenic conditions, the tumor suppressor p53 protein regulates expression of many target genes involved in cell cycle control, DNA repair, senescence and apoptosis. Biological functions of p53 are possible due to its binding to DNA in a sequence-specific or structure-selective manner. The central DNA binding domain (DBD, aa 102–292) is responsible for sequence-specific binding to p53 consensus sequence (p53CON), which consists of two copies of sequence 5′-PuPuPuC(A/T)(T/A)GPyPyPy-3′ separated by 0–13 base pairs [3]. At the C-terminus of p53 protein, there is a second autonomous DNA binding domain (CTDBD, aa 363–382), which is capable of structure-selective recognition of free DNA ends [4], or supercoiled DNA [5]. The capability of p53 to bind non-B DNA structures, e.g., DNA triplex, quadruplex and DNA cruciform, has been described [6,7,8,9]. 

Repetitive sequences containing trinucleotide repeats (TNRs) such as (CTG)n·(CAG)n, (CGG)n·(CCG)n and (GAA)n·(TTC)n represent conformationally flexible genomic DNA sequences with an ability to form non-B DNA structures, especially during DNA metabolism [10,11,12,13]. These types of repeats can form a variety of hairpins, loops, intramolecular triplexes, quadruplexes and slipped-strand structures, which have been implicated in TNR expansion via molecular mechanisms involving DNA replication, recombination or repair [12,13,14]. At least 40 human genetic neurological, neurodegenerative, and neuromuscular diseases are associated with the expansion of TNR sequences [12,15,16]. One example of TNR associated disorder is Huntington’s disease connected to CTG·CAG repeat expansion [17]. Interestingly, high binding affinity of p53 to non-B conformation of CTG·CAG trinucleotide repeats was revealed in previous reports [18,19]. In the case of hairpin formed by CTG-strand, p53 binding improved proportionally to the size of the repeats from one to seven repeats [19]. Another example of TNR associated disease is Friedreich’s ataxia, which is connected with an unstable (GAA)n·(TTC)n trinucleotide repeat expansion in the first intron of the frataxin gene (*FXN*, chromosome 9q13 [12,20,21]). The correlation between the number of (GAA)n·(TTC)n repeats and the Friedreich’s ataxia phenotype severity is well established. Normal chromosomes have 5–30 GAA repeats in which <12 repeats are called short normal alleles and >12 are called long normal alleles [22]. According to experimental model and repeat length, sequences from (GAA)n·(TTC)n repeat were observed to form different non-B DNA structures: slipped DNA with DNA-loops, DNA triplexes, DNA-hairpins or parallel duplexes [23,24,25,26,27].

In this work, we show the binding affinity of p53 for non-B DNA structures formed by GAA, TTC, CTG and CAG trinucleotide repeats. Furthermore, for the first time, we detect p53 interacting with the trinucleotide repeats region of the first intron of frataxin gene folded in vitro into non-B DNA structures and p53 binding to this region in adenocarcinoma cells (MCF7) and Friedreich’s ataxia fibroblast cells (FXN 4654) upon p53 stabilization. These observations provide further evidence that it could be useful to investigate how p53 influences genetic instabilities of TNR sequences that are important for some human diseases.

## 2. Results and Discussion

### 2.1. Interaction of p53 with Non-B DNA Structures Derived from TTC, GAA, CTG and CAG Trinucleotide Repeat Sequences

**Figure 1:**
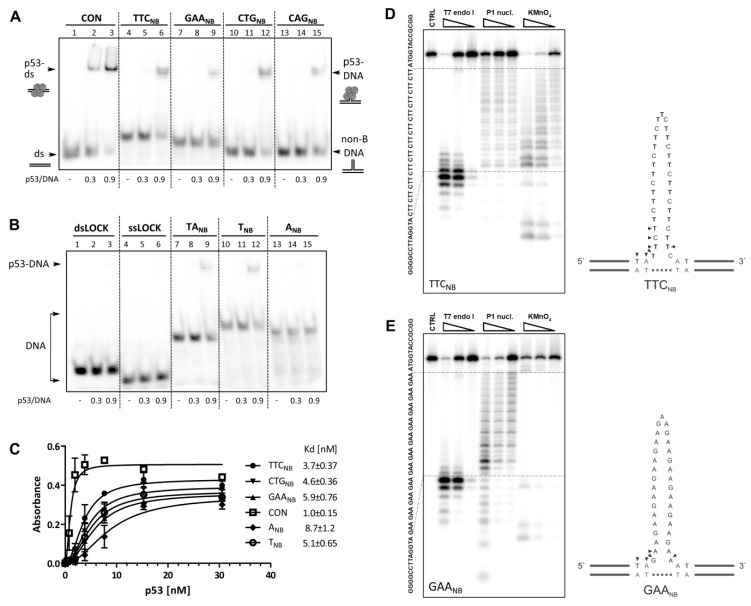
Full length p53 binding to non-B DNA formed by repetitive sequences. Analysis of p53 binding to DNA molecules containing various sequences and structures. DNA sequences are provided in Table S1. In panels (**A**,**B**), radiolabeled oligonucleotides (1 pmol) were bound by p53 in binding buffer in the presence of non-specific DNA competitor (75 ng, BSK/SmaI) and analyzed on a 6% 0.5× TBE polyacrylamide gel. The p53/DNA ratios are given per p53 tetramer. (**A**) DNA molecules are slipped strand mimicking substrates formed by trinucleotide repeat sequences (11 repeats of TTC, GAA, CTG or CAG) and a duplex p53 consensus sequence (CON). (**B**) DNA molecules formed by TA_NB_, T_NB_, A_NB_, and DNA sequence nonspecific for p53 (dsLOCK, ssLOCK). (**C**) p53 binding to biotinylated oligonucleotides by ELISA, portrayed are mean values and SD. The dissociation constants (Kd) calculated per p53 tetramer are indicated. (**D**,**E**) Polyacrylamide gels showing resolved products of TTC_NB_ (**D**) and GAA_NB_ (**E**) enzymatic or chemical cleavage with increasing concentration of T7 endonuclease I (left), P1 nuclease (middle) and potassium permanganate followed by piperidine treatment (right). Right panels show schematic drawings of formed structures. Small arrows indicate preferential cleavage by T7 endonuclease I.

Formation of non-B DNA structures inside TNR sequences is correlated to TNR expansion, that is in turn associated with the progression of a range of human diseases [26]. Previous studies of CTG·CAG tracts (11 repeats) showed that p53 exhibits preferential recognition of hairpin DNA over their linear forms, canonical (CTG·CAG) duplexes or mismatched (CTG·CTG; CAG·CAG) duplexes [18]. In our study, we focused on GAA·TTC TNRs associated with Friedreich’s ataxia, on differences in p53 recognition of the pyrimidine and purine strands of the GAA·TTC repeat and also on comparison to other repeats. We examined TNR sequences containing 11 repetitions, which is in the normal, non-disease range for the Friedreich’s ataxia region [16]. TNR sequences were locked into a non-B DNA form (NB), as previously described for the CTG·CAG repeat [18] and schematically portrayed in Figure 1 and Table S1. This non-B DNA form represents a slipped strand intermediate and may contain several non-B DNA structural motifs: three-way junctions, hairpins, and mismatched duplexes formed by self-annealed TNR strands or loop structures. The binding of p53 protein was examined for each strand of GAA·TTC and CTG·CAG repeats locked within non-B DNA structures, by EMSA (Figure 1A, lanes 4–15) and ELISA (Figure 1C). To compare the amount of p53 bound to Py-strand (TTC_NB_, Figure 1A, lanes 4–6) with protein bound to Pu-strand (GAA_NB_, Figure 1A, lanes 7–9), unbound free DNA (usually forming sharper bands, the intensities of which are easier to evaluate, compared to often more smeary bands of the bound DNA fraction) was monitored. The p53 preference for TTC_NB_ was evident from the amount of free DNA after p53 binding (Figure 1A, compare lane 6 to lane 9). Similarly, as in the case of CAG·CTG repeats and in agreement with a previous study [18], CTG hairpin (CTG_NB_) was preferred substrate for p53 protein over CAG hairpin form (Figure 1A, compare lanes 12 and 15). A relatively strong difference in p53 interaction with Pu- and Py-strands of repeat was also observed with the T_NB_ (Figure 1B, lanes 10–12) and A_NB_ (Figure 1B, lanes 13–15), which were “hairpin-like” structures formed by (T/A)_33_ tracks locked in a loop in the same way as the TNR sequences. Within all of this data, it is important to note that p53 bound the most strongly to the p53 consensus sequence (CON) (Figure 1A lanes 1–3). To better characterize differences in affinities of p53 to different non-B DNA structures and CON, we used ELISA (Figure 1C) followed by quantitation using a specific DO1 antibody as was recently described for p53-telomeric quadruplex DNA-binding [6]. With this system (Figure 1C), we confirmed the EMSA data that p53 binds to TTC_NB_ and T_NB_ with higher affinity than to an equivalent GAA_NB_ and A_NB_. 

To roughly check the proper formation of designed structures, i.e., TNR hairpin or unfolded region within a double-helical DNA, we performed enzymatic footprint of the structures (Figure 1D,E). T7 endonuclease I, specific for mismatched base-pairs and junctions, gave a strong signal at the junction between the TNR part and the locked terminal B-DNA part for both TTC_NB_ and GAA_NB_. Single-strand specific P1 nuclease cleaved at optimal concentration only the TNR part, indicating proper formation of locked B-DNA, but without significant site preference, indicating unfolded TNR part, for both structures. 

In summary, our results show p53 binding to slipped strand mimicking intermediates formed by TNR repeats with preference for TTC repeats over their purine-rich counterparts. Although the p53 binding to some TNRs has been studied previously, here the effects of TTC non-B DNA structure were studied for the first time. We compare p53 binding to TTC with other TNR non-B DNA (GAA, CTG and CAG), with ELISA and EMSA results showing that affinity for the TTC repeat was higher than for the previously published CTG and GAA non-B DNA structures [18,19].

### 2.2. Role of p53 Core and C-Terminal DNA Binding Domains in TNR Non-B DNA Recognition

**Figure 2:**
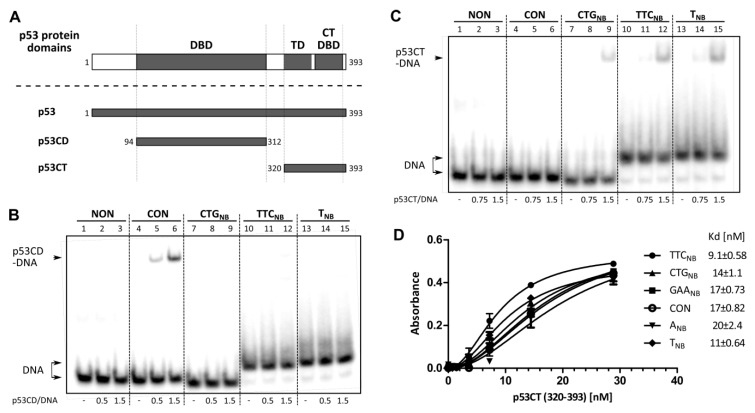
Role of core and C-terminal DNA binding domains in p53 non-B DNA recognition. (**A**) Full-length p53 (p53; DBD—central DNA binding domain; TD—tetramerization domain; CTDBD—C-terminal DNA binding domain) and its deletion variants p53CD (aa 94–312) and p53CT (aa 320–393) were used in this study. (**B**,**C**) EMSA analysis of p53CD (**B**) and p53CT (**C**) binding to CTG_NB_, TTC_NB_, T_NB_, nonspecific (NON) and p53 specific sequence (CON). Radiolabeled oligonucleotides (1 pmol) were bound by p53CD (**B**) or p53CT (**C**) in binding buffer in the presence of non-specific DNA competitor (50 ng BSK/SmaI) and analyzed on polyacrylamide gels (for more details see Figure 1). The p53/DNA ratios are given per p53 tetramer. (**D**) ELISA analysis of p53CT binding to biotinylated oligonucleotides: non-B DNA substrates CTG_NB_, TTC_NB_, GAA_NB_, A_NB_, T_NB_ and p53 specific sequence (CON). Portrayed are mean values and SD. Dissociation constant values (Kd) are calculated per p53CT tetramer in the presence of 2 ng/well of competitor DNA.

To examine the roles of both p53 DNA binding domains (Figure 2A) in p53 TNR slipped-strand mimicking substrate recognition, we analyzed the DNA interactions of isolated p53 core domain (p53CD, aa 94–312, Figure 2B) and p53 C-terminal segment (p53CT aa 320–393; containing p53CTDBD and tetramerization domains, Figure 2C,D). Binding of p53CD and p53CT to CTG_NB_ hairpin, TTC_NB_ and T_NB_ was compared with their binding to the CON (Figure 2B,C, lanes 4–6) and a non-specific DNA sequence (NON, Figure 2B, C lanes 1–3). Importantly, although p53CD was able to form a specific complex with the consensus sequence under the experimental conditions (Figure 2B, lanes 4–6), it was unable to form a stable complex with any of the structure-specific substrates (Figure 2B, lanes 7–15). In contrast to p53CD, binding of p53CT to non-B DNA substrates (CTG hairpin, TTC_NB_ and T-loops) was clearly selective over binding to duplex CON or NON substrates (Figure 2C). The ELISA system (Figure 2D) confirmed p53CT binding to TNR with often higher affinity than to p53CON, it also demonstrated p53CT preference for TTC_NB_ over GAA_NB_. Overall, the C-terminal region of p53, containing CTDBD and tetramerization domain, seems to be chiefly responsible for p53 TTC_NB_ recognition. This corresponds to data acquired by Walter et al. [18], who observed decreased p53 binding to CTG or CAG hairpins upon obstructing the p53 C-terminus by PAb421, an antibody mapping to the C-terminal region.

Our results showing that the C-terminal DNA binding domain with the tetramerization domain is crucial for TNR non-B DNA high affinity binding relate well with our previous studies on other non-B DNA structures, e.g., G-quadruplex [6,9] and T.A.*T* DNA triplex [8].

### 2.3. Binding of Wild-Type p53 to GAA·TTC Sequence in scDNA

**Figure 3:**
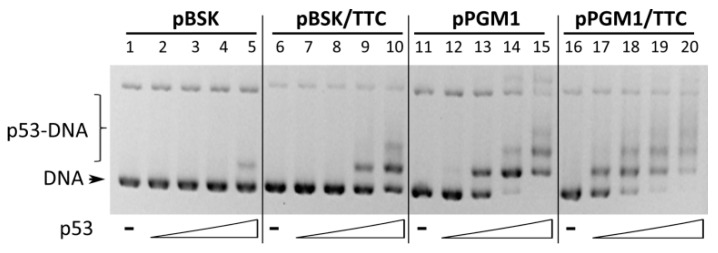
p53 binding to (GAA)_18_·(TTC)_18_ sequence in scDNA. Wtp53 binding to supercoiled plasmids pBSK, pBSK/TTC, pPGM1 and pPGM1/TTC was studied by EMSA in agarose gel. Wtp53 protein was incubated with scDNA (pBSK and pBSK/TTC 100 ng, lanes 1–10), scDNA containing p53CON (pPGM1 and pPGM1/TTC 100 ng, lanes 11–20) in p53/DNA molar ratios 0.5, 1, 1.5 and 2 at 4 °C. EMSA was performed at 4 °C.

To confirm that p53 can bind to TTC repeat sequence also in scDNA an EMSA in agarose gel was performed. Binding of p53 to scDNAs with (pPGM1) or without p53CON (pBSK) was compared with derived plasmids containing (GAA)_18_·(TTC)_18_ sequence (pPGM1/TTC and pBSK/TTC). As expected, binding of p53 to pPGM1 (Figure 3, with p53CON, lanes 11–15) was much stronger than binding to pBSK (Figure 3, lanes 1–5). Introducing TTC sequence did enhance p53 affinity for both plasmids. The used TTC sequence is a relatively weak binding site since it could not by itself (pBSK/TTC) provide the same affinity as an established p53CON (pPGM1), although the affinity will possibly be affected by TNR length. 

The observed binding improvement to p53CON when in combination with TTC (pPGM1/TTC) encourages further exploration into non-B DNA structures as modulators of p53 regulated transcription near p53 consensus sites. In the case of the T.A.*T* type of triplex, we have previously shown that triplex forming sequence in supercoiled plasmid reporter can enhance transcription from a neighbor p53 consensus sequence [8]. In our current work, we detect p53 selective binding to scDNA with TTC repeats indicating that this type of slipped DNA can serve as a non-B DNA binding motif for p53 in complex molecules. 

### 2.4. Analysis of p53 Interaction with TNR Region from the First Frataxin Intron in Cells

**Figure 4:**
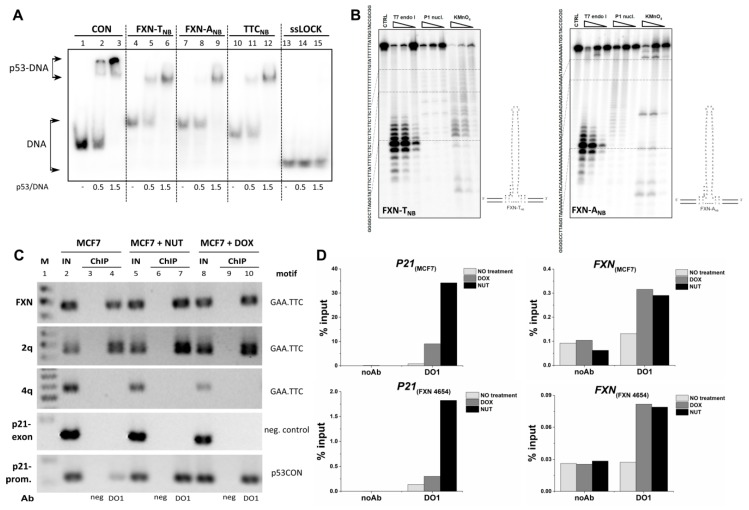
Full length p53 binding to GAA·TTC trinucleotide repeat within the sequence context from the first intron of *FXN*. (**A**) EMSA analysis of p53 binding to repetitive sequences FXN-A_NB_ (GAA rich motif), FXN-T_NB_ (TTC rich motif), TTC_NB_ and nonspecific DNA (ssLOCK) and p53 consensus sequence (CON). Radiolabeled oligonucleotides (1 pmol) were incubated with p53 in binding buffer in the presence of non-specific DNA competitor (50 ng, BSK/SmaI) and analyzed on a polyacrylamide gel. The p53/DNA ratios are given per p53 tetramer. (**B**) Polyacrylamide gels showing resolved products of FXN-T_NB_ (left) and FXN-A_NB_ (right) enzymatic or chemical cleavage with increasing concentration of T7 endonuclease I (left), P1 nuclease (middle) and potassium permanganate followed by piperidine treatment (right). Right panels show schematic drawings of formed structures. Small arrows indicate preferential cleavage by T7 endonuclease I. (**C**) Chromatin immunoprecipitation showing p53 binding to *P21* promoter (positive control) and FXN and 2q regions, which both contain GAA·TTC repeat motifs. Note that p53 binding was not detected on *P21* exon fragment (negative ChIP control) and GAA·TTC repeat containing 4q region. In vitro p53 binding was analyzed in MCF7 cells without and with nutlin-3/doxorubicin 4 hours treatment with DO1 antibody against p53 (lanes 4, 7 and 10), with IgG as negative control ChIP (lanes 3, 6 and9), and with positive input control (1/15 input for ChIP, lanes 2, 5 and 8). (**D**) ChIP data for MCF7 cells treated by 5 μM nutlin-3 (NUT) and 1 μM doxorubicin (DOX) and FXN 4654 cells treated by 5 μM nutlin-3 (NUT) and 0.1 μM doxorubicin (DOX) were acquired by qPCR and presented as percent recovery of input DNA by the specific DO1 antibody. A control without addition of antibody is shown.

Expansion of GAA·TTC repeat in the first intron of the frataxin gene is associated with Friedreich’s ataxia [21]. Using the strategy used to prepare other clamped TNRs, each strand from this region (Table S1) was locked to a non-B DNA structure. Pu-rich strand (FXN-A_NB_) and Py-rich strand (FXN-T_NB_) constructs were used for p53 binding analysis by EMSA. The formation of locked non-B DNA structures of FXN-T_NB_ and FXN-A_NB_ was confirmed by enzymatic cleavage by T7 endonuclease I and P1 nuclease (Figure 4B), similarly as in Section 2.1. The binding of p53 to FXN-T_NB_ (Figure 4A, lanes 4–6) was stronger than to FXN-A_NB_ (Figure 4A, lanes 7–9). The affinity for TTC_NB_ (Figure 4A, lanes 10–12) lies between FXN-T_NB_ and FXN-A_NB_.

To determine if endogenous p53 binds to GAA·TTC expansion region in the first intron of *FXN* we chose this GAA·TTC rich sequence for ChIP analysis in adenocarcinoma cell line MCF7 and Friedreich’s ataxia fibroblast cell line FXN 4654. To detect the p53-DNA binding in cells, we used semiquantitative PCR analysis (Figure 4C) and qPCR (Figure 4D), both employing primers that map the first intron of *FXN* and controls. Detection of p53 binding to *P21* promoter was used as a positive control and binding to *P21* exon was used as negative control (Figure 4C). The GAA·TTC rich frataxin region was bound in the investigated MCF7 cells and we observed increase of p53 binding after p53 stabilization in response to nutlin-3 (NUT) and doxorubicin (DOX) treatment (Figure 4C,D). In case of p53-DNA analysis in Friedreich’s ataxia fibroblast cell line FXN 4654 we were able to detect binding to *p21* promoter region and *FXN* GAA·TTC rich sequence by ChIP-qPCR analysis. Data confirmed the increased region occupancy of *P21* and *FXN* genes after p53 stabilization by NUT and DOX in both tested cell lines (Figure 4D). Taken together, ChIP analysis has confirmed the EMSA results, connecting p53 and TNR associated with Friedreich’s ataxia disease. Interestingly, when probing other genomic GAA·TTC rich regions, p53 bound to the intragenic region on chromosome 2q (Figure 4C, 2q) but not the last intron of RNF150 gene (Figure 4C, 4q). Bioinformatics blast analysis revealed (Figure S1) that both p53 bound regions (FXN and 2q) contain *DNase* I hypersensitivity sites, usually indicating transcriptionally active chromatin. In contrast, 4q GAA·TTC rich region did not contain such sites and was unbound by p53 (Figure 4C). The nucleosome occupancy, methylation status and other factors are probably responsible for the differences in p53 recognition of the three investigated TTC rich segments in human genome.

As for frataxin, a p53-responsive element was recently found in the proximal *FXN* promoter [28,29] and *FXN* was identified as a p53 target gene. The dysfunction of p53 by mutation in cancerous cells was found to decrease the expression of frataxin and deregulate mitochondrial iron homeostasis, similarly to Friedreich’s ataxia disease [29]. However, the previously investigated area did not cover the TNR region, which as we show may add a new regulatory feature to the p53–frataxin interplay.

To examine whether the human *FXN* expression is regulated by p53 in tumor adenocarcinoma cell line (MCF7) and non-tumor cells (fibroblast BJ and Friedreich’s ataxia fibroblast cell line FXN 4654), the cells were treated by nutlin and doxorubicin and RT-qPCR was carried out. As shown in Figure S2A,B in MCF7 cells, the level of *FXN* mRNA and *p21* mRNA markedly increased in dependence on time of treatment (4 h or 16 h) and drugs; doxorubicin strongly increasing oxidative stress response or nutlin primarily stabilizing p53 protein level. The strongest activation of *FXN* expression was observed after 16 h treatment by nutlin. RT-qPCR analysis in non-tumor fibroblast cell lines BJ and FXN 4654 after 4 h treatment showed *FXN* repression in FXN 4654 cells (Figure S2C) and no influence on *FXN* expression in contrast to *p21* after nutlin or doxorubicin treatment in BJ cells (Figure S2D). Our expression analysis of *FXN* in different cell lines showed that further studies are necessary to understand the p53 role in frataxin biology. Frataxin repression after doxorubicin (oxidative stress inducer) treatment was observed previously in normal cells (cardiomyoblasts) [30].

The role of oxidative stress in Friedrich ataxia’s cells [31] and p53 function in stress response have to be considered. The reduction of frataxin expression is associated with increased oxidative stress and cell damage, accumulation of iron in mitochondria [32]. These factors can strongly influence p53 function by oxidative damage of protein. The central domain of p53 protein is sensitive to cysteine oxidation which leads to reduction or abolishment of sequence specific interaction with p53 consensus element [33] but p53 non-B DNA structure recognition is partially maintained for oxidized p53 as was observed for G-quadruplexes [6] or supercoiled DNA [33]. We do show an increase in p53 occupancy on frataxin TNR regions when treated with oxidative stress inducing agent doxorubicin. The C-terminal domain directed binding may represent a potential mechanism by which p53 could regulate frataxin under oxidative stress. 

### 2.5. Future Prospects of p53 and TNR Non-B DNA Structures

In cancer genomes, (GAA)_n_ repeats, together with (AT)_n_, and (GAAA)_n_, constitute the most frequent repeats at translocation breakpoints, which represent an intrinsic risk factor for genomic rearrangements [34]. Interestingly, unlike CNG repeats, GAA repeats display unique behavior during TNR repeat expansion: a) strict strand orientation dependence of the replication stalling; b) GAA repeats expansion occurs once they reach a certain threshold [35]. As p53 is involved as a transcription factor and also by direct interactions with several relevant proteins in processes that might be important in TNR stability such as homologous recombination, mismatch repair, replication [2,36,37], it is a future prospect to investigate p53 influence on TNR stability.

The GAA triple repeat expansion in Friedreich’s ataxia is directly responsible for the transcriptional silencing of frataxin gene [21]. Nowadays two model potential mechanisms are considered to be responsible for this reduction of frataxin expression: a) the formation of non-B DNA structures (including RNA_DNA hybrid non-B structures or GAA triplexes) and b) heterochromatin formation [32]. Inhibition of the frataxin transcription process by non-B DNA conformation was observed *in vitro* and *in vivo* [32,38]. 

The significance of the observed p53 preference for Py-rich TNR non-B DNA structures is proposed to be associated with a mechanism of gene silencing in Friedreich’s ataxia (Figure S3). The transcriptional block of Pol II on long GAA·TTC repeats occurs via the TTC strand [32]. So far, we can only speculate if the recently observed p53 regulation of DNA-binding function of Pol II [39], can also influence Pol II transcription of frataxin gene. The transcriptional weak spot represented by the extruded TTC strand is however preferentially recognized by p53, as is shown in the current study (Figure S3). The triplex DNA formation is another proposed mechanism of Pol II transcriptional block on frataxin TNR. In our previous work, we observed preferential p53 recognition of DNA T.A.*T* triplex. We used the luciferase reporter assay to detect the effect of a T.A.*T* triplex on p53 driven transcription [8]. We observed T.A.*T* triplex dependent enhancement of reporter plasmid activation with p53CON sequence and triplex forming sequence by luciferase reporter assay. We also observed that the same sequence can block transcription of reporter plasmid if it contains only T.A*.T* triplex motif in supercoiled reporter [8]. Any future reporter assays with TNR have to take into account the influence of DNA topology because DNA supercoiling is a necessary condition for non-B DNA formation [8]. The requirement for a threshold level of DNA supercoiling may be the reason why the study of the influence of CTG·CAG repeat track on a linear luciferase reporter did not show profound effect for p53 [18].

Further studies are needed to understand the precise function of p53 TNR non-B DNA recognition in relation to the development of Friedreich’s ataxia or other diseases coupled with TNR expansion.

The ability of p53 to interact with two DNA binding domains makes the protein very flexible in terms of its physiological functions. The C-terminal DNA binding domain is mainly responsible for high affinity non-B DNA recognition by p53 [6,8,9] and, for the first time, our work shows that this domain is crucial for TNR non-B DNA recognition. It is expected that the ability of p53 to recognize TNR non-B DNA is conserved in the majority of hot spot cancer mutant p53 proteins [40]. About 90% of *TP53* mutations are localized to the central part of gene, the majority of mutant p53 will still have functional C-terminus, so they will be able to recognize non-B DNA structures formed by TNR, as already shown for hot spot R273H mutant p53 to recognize CTG non-B DNA structures [18], which may be a reason why p53 status was not associated with Friedreich’s ataxia. In contrast, C-terminal mutants in lysine residues are expected to not recognize TNR non-B similarly to truncated mutant p53 without 30 last amino acids [6,9,41]. 

In summary, we show that non-B DNA structures formed by TNR (TTC, GAA, CTG, CAG) and simple T-repeat are recognized by p53. Moreover, p53 prefers non-B DNA structures formed by the pyrimidine-rich strands of the investigated repetitive sequences and that the intact C-terminus is responsible for high p53 affinity to TNR non-B DNA structures.

## 3. Materials and Methods

### 3.1. Oligonucleotides

The sequences of oligonucleotides used in this study are presented in Table S1, and these were purchased from Sigma-Aldrich (Praha, Czech Republic). DNA hairpin or DNA loop structures were prepared as described in [18,19]. Briefly, Lock-F oligonucleotide was mixed with the repeat containing complementary strands in buffer (10 mM Tris-HCl, pH 8; 0.1 mM EDTA and 50 mM KCl) and placed in a heating block at 95 °C for 5 min. DNA substrates were allowed to cool slowly and anneal for a minimum of 3 hours. For EMSA Lock-F was 5′-labeled by T4-DNA polynucleotide kinase (NEB) with ^32^P-gamma ATP. For ELISA Lock-F was biotinylated at the 3′ end.

### 3.2. Recombinant Plasmids

Plasmids allowing controlled expression of human p53 proteins pT7-7wtp53 (full length wild type p53, aa 1–393), pGEX-4Tp53CD (GST-p53CD, aa 94–312) and pGEX-2TKp53CT (GST-p53CT, aa 320–393) were described previously [5]. Nonspecific competitor (pBSK/SmaI) was prepared by SmaI restriction enzyme (Takara, Japan) cleavage of pBluescript SK II- (pBSK, Stratagene). pPGM1 was derived from pBSK by cloning 20bp p53 consensus sequence into HindIII restriction site. Plasmids pBSK/TTC and pPGM1/TTC were prepared by cloning into the EcoRI/SmaI restriction site, establishing a continual 18× GAA·TTC repetition. All plasmids were isolated from *E. coli* bacterial strain TOP10 (Stratagene) and verified by sequencing.

### 3.3. Purification of p53 Recombinant Proteins

Full length p53 and isolated DNA binding domains (p53CD (aa 94–312) and p53CT (aa 320–393)) were purified via their GST tag according to a protocol described previously [5,40].

### 3.4. EMSA in Polyacrylamide Gels

Electrophoretic mobility shift assays were performed with ^32^P-radiolabeled oligonucleotide probes (1 pmol) mixed with p53 proteins and incubated in binding buffer (5 mM Tris-HCl, pH 7.6; 0.5 mM EDTA; 0.5 mM DTT; 0.01% Triton-X100 and 50 mM KCl) in the presence of 50 or 75 ng pBSK/SmaI DNA competitor for 15 min on ice to reach equilibrium. Samples were loaded onto a 6% polyacrylamide gel containing 0.5× Tris-borate-EDTA (TBE) buffer. After 30 min electrophoresis (at 6 V/cm) the gels were dried, and DNA was detected by autoradiography using Typhoon FLA 9000 (GE Healthcare). Representative gels from at least three repetitions are shown.

### 3.5. EMSA in Agarose Gels

Wtp53 protein was incubated with 100 ng scDNA in p53/DNA molar ratios 0.5–2 in binding buffer (5 mM Tris-HCl, pH 7.6; 0.5 mM EDTA; 0.5 mM DTT; 0.01% Triton-X100 and 50 mM KCl) for 15 min at 4 °C. Samples were loaded onto a 1% agarose gel containing 0.33× Tris-borate-EDTA (TBE) buffer. After 5 h electrophoresis (at 4–6 V/cm) agarose gels were stained with ethidium bromide (EtBr) and photographed. Representative gel from at least three repetitions is shown.

### 3.6. ELISA

A 96-well Immuno Plate (SPL LIFE SCIENCES) was coated overnight at 37 °C with 5 ug/mL streptavidin (PROSPEC) in 1× PBS (137 mM NaCl; 2.7 mM KCl; 8 mM Na_2_HPO_4_ and 2 mM KH_2_PO_4_) (50 μL/well). The plate was rinsed with 1× PBS (200 μL/well) and blocked for unspecific binding by 3% BSA (Sigma-Aldrich) in 1× PBS (2 h, room temperature, 200 μL/well). Next, 0.3 pmol of biotinylated DNA oligonucleotides (50 μL/well) in 1× TTKD (5 mM Tris-HCl, pH 7.6; 0.01% Triton-X100 and 50 mM KCl; 0.5 mM DTT) were bound to the plate (1 h, room temperature). The plate was rinsed with 1× PBST (1× PBS; 0.05% Tween) (200 μL/well) and then pre-incubated protein–primary antibody mixes (in 2:1 Ab:protein molar ratio in 1× TTKD) were added (50 μL/well). Experiments were performed with p53 and p53CT; in experiments with p53CT, 2 ng of competitor (pBSK/SmaI) was added per well, more details in [6,8]. The plate was incubated 20 min at 4 °C and rinsed in 1× PBST and three times in 1× PBS + 0.5% BSA (200 μL/well). Secondary HRP-labeled antibody was incubated on the ELISA plate for 30 min at 4 °C (50 μL/well). The plate was washed in 1× PBST and three times in 1× PBS + 0.5% BSA (200 μL/well), after which 50 μL/well of TMB substrate was added. The TMB (3,3’,5,5’-Tetramethylbenzidine, Sigma) was dissolved to 1 mg/mL in DMSO (dimethyl sulfoxide). The working solution was made by diluting 0.6 mL of the DMSO stock with 5.4 mL phosphate–citrate buffer, pH 5.0, and then adding 1.2 μL of fresh 30% hydrogen peroxide. Absorbance was measured at 370 nm on Synergy H1 (BioTek) and evaluated in GraphPad Prism using hyperbolic or “Hill” equation fittings. Dissociation constants (Kd) were obtained from at least three independent measurements and the values are given per tetramer for full length p53 and p53CT.

### 3.7. Chromatin Immunoprecipitation

Nutlin-3 and doxorubicin were investigated to stabilize the endogenous p53 and subjected to chromatin immunoprecipitation (ChIP) assays as previously described in [8]. Purified monoclonal DO1 antibody and IgG (negative control) were used for ChIP. The PCR was performed using the primers targeting the expected p53 binding site referred to as *P21* promoter (p21-chipF: GCACTCTTGTCCCCCAGGCT; p21-chipR: GGTCTCCTGTCTCCTACCAT) [8], the GAA·TTC loci of the first intron of the frataxin gene (FXNchip_F1721: GAAAGAGGCAGGCCACGTCCAAG; FXNchip_R2170C: CATTGGGCGATCTTGGCTTAATGC) and another GAA·TTC rich regions: intragenic region on chromosome 2q (2q36F: AAAAGGGCAACATGAACTACTG, 2q36R: ACTTTTGCCCAGAGATAACTC) and intronic region of the *RNF150* gene on 4q chromosome (4q31F: AGCTTTGCTAGGAACCAATATG; 4q31R: TGTCAGATAACTGAACTTGTAC) described in [42] and *P21* exon region (p21-F: CCTCAAATCGTCCAGCGACCTT; p-21-R: CATTGTGGGAGGAGCTGTGAAA).

Friedreich’s ataxia skin fibroblast cell line (FXN 4654, ~190 and ~500 GAA repeats, Kerafast) was grown in DMEM medium supplemented with 15% FBS, penicillin/streptomycin (BioSera) and 1% MEM Non-essential Amino Acid Solution (100×) (Sigma-Aldrich) and incubated at 37 °C with 5% CO_2_. Cells were treated for 4 hours with nutlin-3 (5 μM) and doxorubicin (0.1 μM).

Human breast adenocarcinoma cell line MCF7 (HTB-22, ATCC) was grown in DMEM medium supplemented with 5% FBS and penicillin/streptomycin (Biosera). The medium was supplemented 24 hours before the treatment with higher concentration of FBS (10%) and treated for 4 hours with nutlin-3 (5 μM) and doxorubicin (1 μM). PCR-based analysis of ChIP DNA was carried out in triplicates for 25 or 30 cycles, with the following conditions (94 °C 2 min; 94 °C 1 min; 65 °C 1 min 25–30×; 72 °C 1 min; 72 °C 10 min). qPCR was performed on Rotor-Gene 6000 (Corbett Research) in the standard program (15 min at 95 °C; 15 s, 95 °C; 30 s, 60 °C; 20 s, 72 °C; 10 s, 74 °C; 50 cycles) using EvaGreen (Solis Biodyne) fluorescent dye.

Number of TTC repeat in MCF7 (eight times) and FXN 4654 (~190 and ~500 GAA repeats) cells was controlled by PCR fragment analysis for ChIP primers and by sequences of this fragment in case of MCF7 cells. 

### 3.8. Expression Analysis

Cells (normal fibroblast skin cell line BJ (ATCC^®^ CRL-2522^™^), FXN 4654 and MCF7) were harvested using trypsin (Biosera) and seeded into 6 cm tissue culture dishes (TPP). After 24 hours cells were treated with 0.1 μM doxorubicin or 1 μM Nutlin-3 for 4 hours. For RT-qPCR analysis, total RNA was isolated by applying NucleoSpin RNA II (MachereyNagel, according to the manufactures instruction) (Düren, Germany) and 2 μg of RNA were reverse transcribed using the High Capacity RT kit (Applied Biosystems, according to manufactures protocol) (Brno, Czech Republic). PCR was performed using the EvaGreen (Solis Biodyne) fluorescent dye in the standard program (15 min 95 °C; 15 s 95 °C, 30 s 60 °C, 20 s 72 °C, 10 s 74 °C; 50 cycles) running in the RotorGene 6000 (Corbett Research). PCR reactions for each sample were repeated in triplicates. The GAPDH reference gene was used as endogenous control. An absolute quantification of mRNA levels was done and relatively related to control (with no treatment). The following primer sets were used: GADPH-QF15: ACAACTTTGGTATCGTGGAAGG; GADPH-QR15: GCCATCACGCCACAGTTTC, FXN-F: CAGAGGAAACGCTGGGACTCT; FXN-R: AGCCAGATTTGCTTGTTTGG; p21-F: CCTCAAATCGTCCAGCGACCTT; p-21-R: CATTGTGGGAGGAGCTGTGAAA.

### 3.9. Enzymatic and Chemical Footprinting

TNR containing sequences were radiolabeled using ^32^P-gamma ATP and T4 polynucleotide kinase. Labelled sequences (0.4 pmol) were heated for 5 min at 90 °C with 0.5 pmol of lock sequence in 10 mM Tris-HCl, pH 8 with 50 mM KCl and cooled to room temperature. Formed structures were treated at 23 °C with decreasing amounts of T7 endonuclease I (10 to 2 to 0.4 units) for 30 min, with 10 mM MgCl_2_, P1 nuclease (1 to 0.2 to 0.04 units) for 30 min, with 1 mM ZnCl_2_ or potassium permanganate (2 to 0.4 to 0.08 mM) for 10 min. Potassium permanganate reaction was stopped by β-mercaptoethanol and oligonucleotides were ethanol-acetate precipitated, treated for 30 min by 1 M piperidine at 90 °C and piperidine was removed by extensive lyophilization. DNA from nuclease reactions was phenol/chloroform extracted and ethanol–acetate precipitated. Thirty kcpm of radiolabeled DNA were dissolved in formamide loading dye, denatured and loaded on a pre-heated 20% polyacrylamide denaturing gel with 7 M urea and 1× TBE. Gels were run for 90 min at 45 W in 1× TBE. Results were visualized by a 2 hour exposition to a PhosphorImager screen and scanned on a Typhoon FLA9000 scanner (GE Healthcare Life Sciences).

## Supplementary Materials

The following are available online, Figure S1: Blat analysis of GAA·TTC rich regions used for ChIP, Table S1: DNA oligonucleotide sequences used in experiments.
