# Supplementary material for: p53 Binds Preferentially to Non-B DNA Structures Formed by the Pyrimidine-Rich Strands of GAA·TTC Trinucleotide Repeats Associated with Friedreich’s Ataxia
Source: Molecules. 2019 May 31;24(11):2078. doi: 10.3390/molecules24112078 (PMC6600395; doi:10.3390/molecules24112078)
Supplement: Supplementary file 1 [file molecules-24-02078-s001.zip › FigureS1-3.pdf]

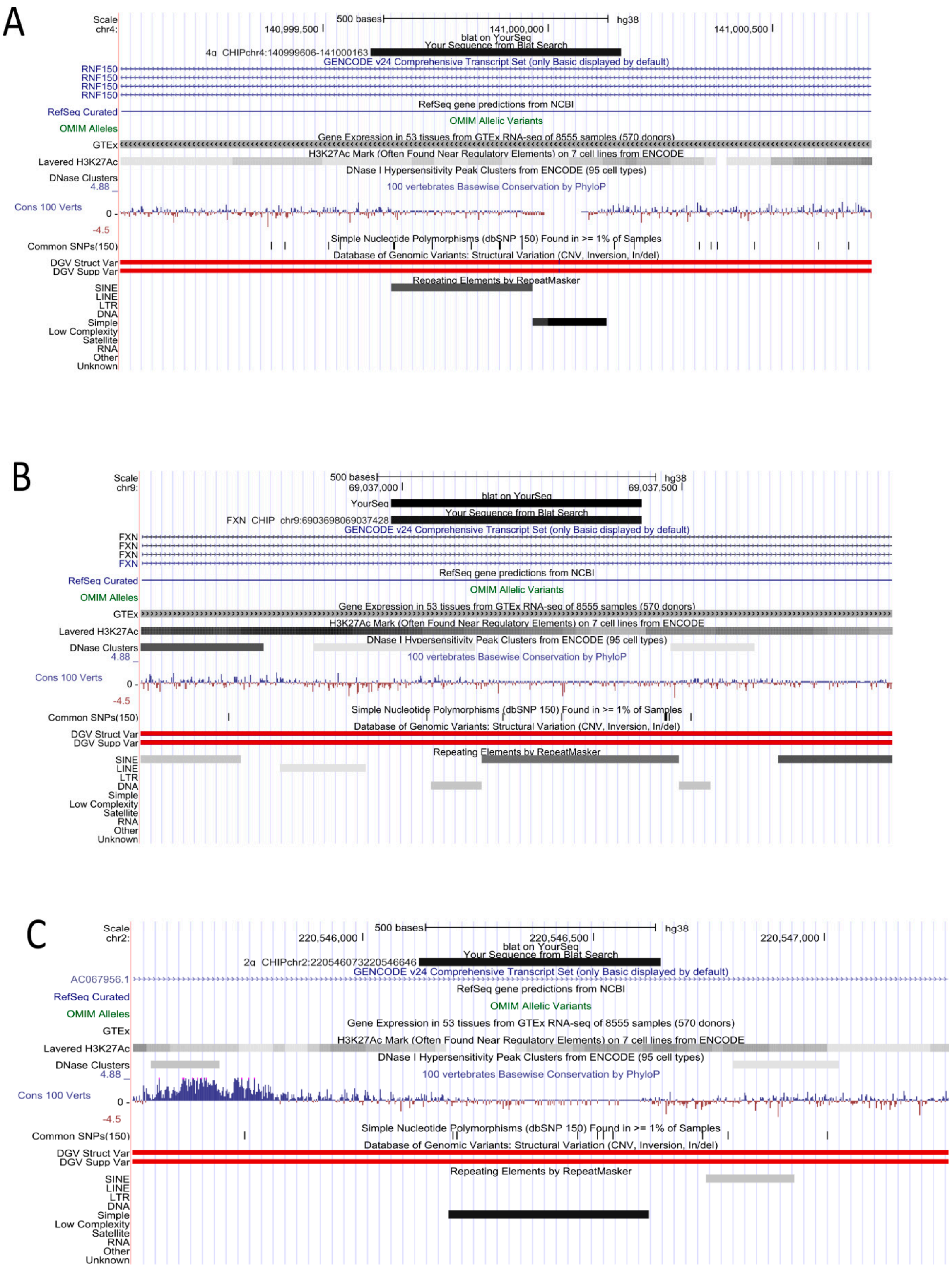

Figure S1

Figure 1. Blat analysis of GAA.TTC rich regions used for ChIP: (A) 4q region, from the last intron of RNF150 gene; (B) FXN-ChIP, from the first intron of *frataxin*; (C) 2q region: intragenic region. Analyzed ChIP sequences differ in presence of DNase clusters (DNase I hypersensitive sites).

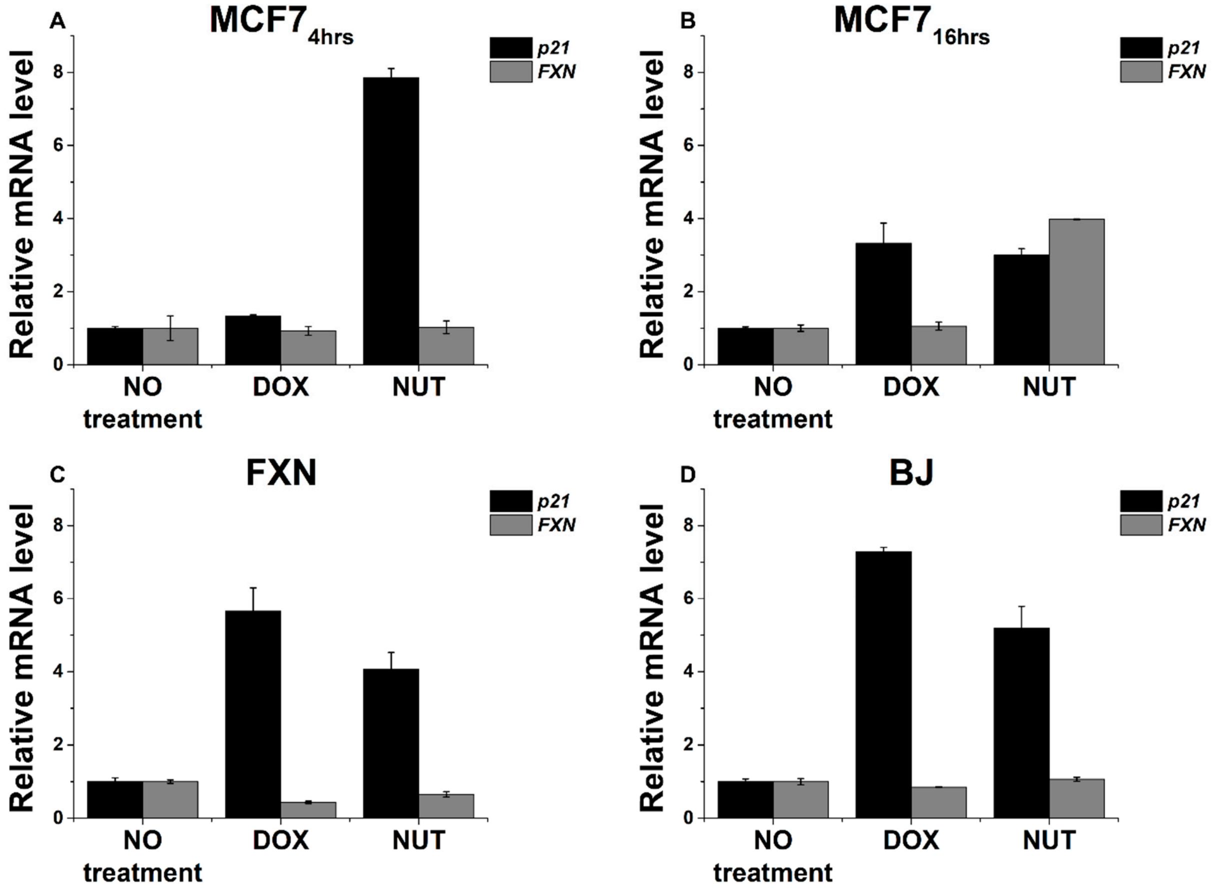

Figure 2. Relative mRNA levels for *P21* and *FXN* in MCF7, FXN 4654 and BJ cells without and with nutlin-3/doxorubicin treatment (4 hours).

3 Expression analysis

Cells were haverested using trypsin (Biosera) and seeded into the 6 cm tissue culture dish (TPP). After 24 hours cells were treated with 0,1 μM doxorubicine and 1 μM Nutlin-3 for 4 hours. For qRT-PCR analysis, total RNA was isolated by applying NucleoSpin RNA II (MachereyNagel - according to the manufactures instruction) and 2 μg of RNA were reverse transcribed using the High Capacity RT kit (Applied Biosystems - according to manufactures protocol). PCR was performed using the EvaGreen (Solis Biodyne) fluorescent dye in the standard program (15 min 95 °C; 15 s 95 °C, 30 s 60 °C, 20 s 72 °C, 10 s 74 °C; 50 cycles) running in the RotorGene 6000 (Corbett Research). PCR reactions for each sample were repeated in triplicates. The GAPDH reference gene was used as endogenous control. An absolute quantification of mRNA levels was done and relatively related to control (with no treatment). The following primer sets were used: GADPH-QF15: ACAACTTTGGTATCGTGGAAGG; GADPH-QR15: GCCATCACGCCACAGTTTC,FXN-F:CAGAGGAAACGCTGGGACTCT; FXN-R: AGCCAGATTTGCTTGTTTGG; p21-F: CCTCAAATCGTCCAGCGACCTT; p-21-R: CATTGTGGGAGGAGCTGTGAAA.

Figure 3. Hypothetical role of p53 in GAA.TTC repeat-mediated gene silencing in FRDA. *Blue*, GAA strand; *yellow*, TTC strand; *red*, Pol II: mRNA polymerase; *orange*, DNA_RNA hybrid; *green*, transcribed RNA; *cyan*, p53 tetramer. (A) Normal *frataxin* transcription. In the presence of short GAA repeat, Pol II normally transcribes *frataxin* (RNA in *green*). (B) p53 binding to DNA triplex in FRDA. In the presence of a long pathological $(GAA)_n$ repeat expansion, p53 can potentially stabilize DNA triplex. (C) Role of p53 in the transcriptional block in FRDA. Once the triplex is formed, a block of the transcription possibly occurs (distal pause) due to p53 blocking the TTC from Pol II. (D) DNA_RNA hybrid formation in FRDA. Picture was modified according to [1].

1. Marmolino, D. and F. Acquaviva, *Friedreich's Ataxia: from the (GAA)n repeat mediated silencing to new promising molecules for therapy*. Cerebellum, 2009. **8**(3), 245-59 DOI: 10.1007/s12311-008-0084-2.
