# Supplementary material for: p53 Binds Preferentially to Non-B DNA Structures Formed by the Pyrimidine-Rich Strands of GAA·TTC Trinucleotide Repeats Associated with Friedreich’s Ataxia
Source: Molecules. 2019 May 31;24(11):2078. doi: 10.3390/molecules24112078 (PMC6600395; doi:10.3390/molecules24112078)
Supplement: Supplementary file 1 [file Table S1.pdf › Table S1.pdf]

**Table S1** DNA oligonucleotide sequences used in experiments. Sequences in red do not have a complementary sequence on the annealed strand, so are expected to form non-B DNA structures (NB).

| Name | Sequence | Schematic view of structure |
|---|---|---|
| Lock -F[a] | 5´ CCGCGGTACCATTACCTAAGGCCCCAAC 3´ | |
| Lock -R | 5´ GTTGGGGCCTTAGGTAATGGTACCGCGG 3´ | |
| $CTG_{NB}$ | 5´ GGGGCCTTAGGTACTGCTGCTGCTGCTGCTGCTGCTGCTGCTGATGGTACCGCGG 3´<br>3´ CAACCCCGGAATCCAT==============================TACCATGGCGCC 5´ | |
| $CAG_{NB}$ | 5´ GGGGCCTTAGGTACAGCAGCAGCAGCAGCAGCAGCAGCAGCAGATGGTACCGCGG 3´<br>3´ CAACCCCGGAATCCAT==============================TACCATGGCGCC 5´ | |
| $GAA_{NB}$ | 5´ GGGGCCTTAGGTAGAAGAAGAAGAAGAAGAAGAAGAAGAAGAAATGGTACCGCGG 3´<br>3´ CAACCCCGGAATCCAT==============================TACCATGGCGCC 5´ | |
| $TTC_{NB}$ | 5´ GGGGCCTTAGGTATTCTTCTTCTTCTTCTTCTTCTTCTTCTTCATGGTACCGCGG 3´<br>3´ CAACCCCGGAATCCAT==============================TACCATGGCGCC 5´ | |
| $CGG_{NB}$ | 5´ GGGGCCTTAGGTACGGCGGCGGCGGCGGCGGCGGCGGCGGCGGATGGTACCGCGG 3´<br>3´ CAACCCCGGAATCCAT==============================TACCATGGCGCC 5´ | |
| $TA_{NB}$ | 5´ GGGGCCTTAGGTATATATATATATATATATATATATATATATATAATGGTACCGCGG 3´<br>3´ CAACCCCGGAATCCAT==============================TACCATGGCGCC 5´ | |
| $T_{NB}$ | 5´ GGGGCCTTAGGTATTTTTTTTTTTTTTTTTTTTTTTTTTTTTTATGGTACCGCGG 3´<br>3´ CAACCCCGGAATCCAT==============================TACCATGGCGCC 5´ | |
| $A_{NB}$ | 5´ GGGGCCTTAGGTAAAAAAAAAAAAAAAAAAAAAAAAAAAAAAAATGGTACCGCGG 3´<br>3´ CAACCCCGGAATCCAT==============================TACCATGGCGCC 5´ | |
| FXN-$T_{NB}$ | 5´ GGGGCCTTAGGTATTTTCTTTATTTTCTTCTTCTTCTTCTTTTTTTTTTTTTTTTTGTATTTTTTTATGGTACCGCGG 3´<br>3´ CAACCCCGGAATCCAT=====================================================TACCATGGCGCC 5´ | |
| FXN-$A_{NB}$ | 5´ GGGGCCTTAGGTAAAAAAATACAAAAAAAAAAAAAAAAAGAAGAAGAAGAAGAAAATAAAGAAAAATGGTACCGCGG 3´<br>3´ CAACCCCGGAATCCAT=====================================================TACCATGGCGCC 5´ | |
| CON-F | 5´ GACGGTATCGATAAGAGACATGCCTAGACATGCCTCTTGATATCGAATTC 3´ | |
| CON-R[a] | 5´ GAATTCGATATCAAGAGGCATGTCTAGGCATGTCTCTTATCGATACCGTC 3´ | |
| NON-F | 5´ ACCAGAGCTGATGGTATCCTAAGTTGACGACCCCGAGGGTGCCGCAAGGA 3´ | |
| NON-R | 5´ TCCTTGCGGCACCCTCGGGGTCGTCAACTTAGGATACCATCAGCTCTGGT 3´ | |

[a] Substrates for ELISA were biotinylated at 3´ end.
